# Comparative property investigation of raw and treated coconut shell biomass for potential polymer composite application

**DOI:** 10.1016/j.heliyon.2024.e40704

**Published:** 2024-11-26

**Authors:** Dennis O. Bichang'a, Isiaka O. Oladele, Oladunni O. Alabi, Fatai O. Aramide, Olasunkanmi Oluseye, Sunday G. Borisade, David N. Githinji, Mike O. Ojemaye

**Affiliations:** a Department of Mechanical Engineering, Kirinyaga University, P. O. Box 143-10300, Kerugoya, Kenya; b Department of Metallurgical and Materials Engineering, Federal University of Technology, Akure, PMB 704, Ondo State, Nigeria; c Department of Chemistry, Federal University of Technology, Akure, PMB 704, Ondo State, Nigeria; d Department of Materials and Metallurgical Engineering, Federal University Oye Ekiti, Nigeria; e Department of Manufacturing, Industrial and Textile Engineering, Moi University, P. O. Box 3900, Eldoret, Kenya; f Department of Chemistry, University of Fort Hare, Alice, 5700, Eastern Cape, X1314, South Africa

**Keywords:** *Coconut shell*, *alkali and bleaching treatments*, *Crystallinity index*, *Thermal stability*, *Sustainable material*

## Abstract

The use of environmentally friendly materials for industrial applications has increased tremendously in the past decades due to environmental concerns associated with using synthetic materials. The present comparative investigation studied the properties of raw and chemically-treated coconut shell biomass for possible polymeric composite applications. The coconut shell biomass was treated with alkali (NaOH), bleaching and combined NaOH-bleaching solutions and investigated the surface morphology, chemical transformations, and thermal stability. Untreated and chemically modified coconut shell biomass was characterized through the determination of chemical constituents, X-ray diffraction (XRD), Fourier Transform infrared spectroscopy (FTIR), thermogravimetric (TGA), and morphological analyses.

Chemically treated coconut shell biomass reported a significant increase in cellulose constituents, reaching 74.72% for combined NaOH-bleach treated samples with accompanying reductions in lignin and hemicellulose, as confirmed by FTIR spectroscopy. Further, the study reported an increase in crystallinity index with chemical treatment. For instance, combined NaOH-bleach treatment reported a maximum crystallinity index of 80.29% compared to 44.82% for untreated biomass. Alkali treatment improved thermal stability as indicated by an increase in the onset temperature of degradation to 255°C from 250°C for raw samples. Post-treatment, improved surface purity and roughness were observed, indicating enhanced fibre/matrix interlocking during composite fabrication. Moreover, combined NaOH-bleaching treatment exhibited enhanced surface hydrophobicity, as indicated by a maximum C/O ratio of 0.93 compared to 0.64 for untreated samples. In conclusion, combined NaOH-bleaching treatment significantly improved the chemical, structural and morphological properties of coconut shell biomass, suggesting its potential for developing low-cost, lightweight, renewable, and sustainable composite materials.

## Graphical abstract

**Figure:**
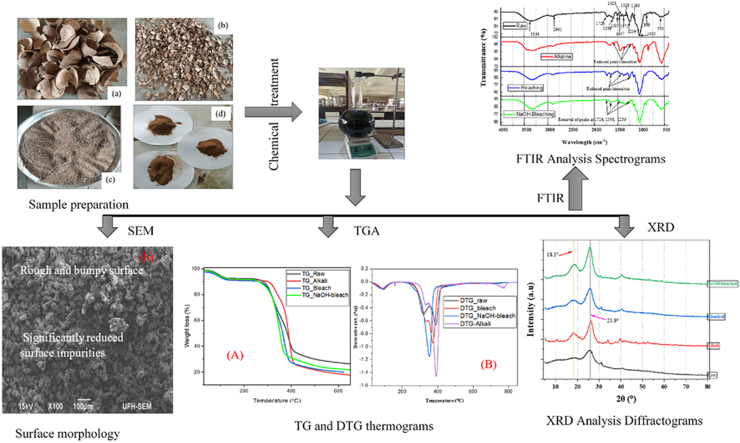

## 1 Introduction

The world is currently grappling with challenges of global warming, environmental pollution, and energy crisis due to overdependence on non-renewable petroleum-based raw materials [1]. This has necessitated the need for eco-friendly and sustainable materials for diverse engineering applications such as aerospace, automobile, sports, marine, and packaging, among others. Natural filler-based polymer materials are currently used in these application areas due to their lightweight, high corrosion resistance, nonbiodegradability, sustainability, nontoxicity, and low-cost attributes compared to synthetic counterparts [2,3]. As such, reinforcements from natural sources have recently attracted increased research attention as an environmentally friendly and inexpensive substitute for synthetic reinforcements in composite development. Unlike synthetic materials, natural materials offer the benefits of lightweight, low-cost, renewability, biodegradability, high abundance, high specific stiffness, and nontoxicity [4,5].

In recent years, natural lignocellulosic materials, such as hemp, bamboo, flax, sisal, etc., have become the most significant reinforcing materials in polymer matrices. Using these wood-based raw materials involves cutting plant fibres, contributing to the triple planetary crisis, namely loss of biodiversity, pollution, and climate change [6,7]. With increasing application areas of polymer composites, the consumption of wood-based raw materials is expected to increase. Therefore, substitutions for wood-based raw materials are inevitably needed. Recently, there has been a growing research interest in agricultural residues as substitutes for wood-based raw materials. Of the various agricultural residues, such as bagasse, barley, wheat, rice, sorghum, etc., coconut shell presents interesting properties in biodegradable polymer composites due to its good thermal stability [8].

Coconut is an important global crop in Southeast Asia: Indonesia, the Philippines, and Malaysia. The cultivation of this plant has spread to tropical regions of the world, such as South America and Africa. The coconut shell is a protective layer of the coconut fruit that comprises endocarp, endosperm, and mesocarp layers [9]. In 2017, Nigeria produced 267,520 metric tons of coconut fruits, translating to more than 40,000 metric tons of coconut shell agricultural residues. However, more than half of the coconut shell waste generated in third-world countries, such as Nigeria, is used for open-burning charcoal production as well as dumped in landfills, causing air and land pollution, respectively, due to the lack of technological capacity for sustainable and eco-friendly utilization of these wastes [10]. Further, improper coconut waste management causes water and soil contamination, making it a global issue that needs a concerted approach. Several studies have investigated the possibility of using coconut shell agricultural residues for charcoal production [11], sustainable biofuel production [10,12,13], concrete production [14], and particle filler in eco-composite materials [8,[15], [16], [17], [18]]. Research on coconut shell waste could provide solutions to the global energy crisis and environmental issues besides boosting the agricultural economy [19,20].

Natural cellulosic materials have hydrophilic lignocellulosic molecules and low thermal stability, rendering them incompatible with polymeric matrix [21]. This has necessistated the need for surface modification to enhance fibre/matrix compatibility, hence interfacial bonding strength. With the increased potential of natural material-based composites in diverse industrial applications, surface modification of natural fibers and particles is becoming a major research area. Generally, silane, alkali, peroxide, bleaching, acetylation, and benzoylation treatments of natural fibers have been widely investigated so far [[22], [23], [24], [25], [26]]. For instance, a comparative study on the impact of the alkali and bleaching treatments of natural cellulosic fibres from *Juncus Effesus* L plant revealed that bleach-treated fibres exhibited enhanced properties and relatively high cellulosic content due to significant removal of amorphous constituents, mainly hemicellulose and lignin [27]. Similarly, characterization results of *Bauhinia Variegata* natural fibre subjected to alkali and bleaching treatments reported improved tensile strength and Young's Modulus as well as reduced hydrophilic nature of the fibre with chemical modification and enhanced natural fibre/matrix interfacial bonding strength [24].

Although chemical modification of cellulosic fibres using different chemical solutions presents enhanced fibre properties, the adverse environmental impact of treatment solution remains a major challenge. For instance, a study on environmentally sustainable chemical treatment of jute, flax, and hemp cellulosic fibres using NaOH and sodium bicarbonate (NaHCO_3_) compared the pH values of residual solutions after the treatment to establish the acidity levels. The pH value of the NaOH treatment solution was found to be strongly alkaline (pH 11–13), indicating harmful ecological impacts in the disposing environment. On the other hand, the pH value of NaHCO_3_ was found to be neutral (pH 7–8), indicating that the solution is not harmful to the environment [28]. The study recommended using NaHCO_3_ as an environmentally friendly treatment of natural fibres. Therefore, studies on chemical treatment of natural fibres should consider neutralizing the treatment solution to attain a neutral pH prior to chemical effluent disposal to minimize the environmental impacts of the solutions in the disposing area.

Previous studies have explored the influence of different chemical treatments on the microstructural properties of coconut shell biomass for polymer composite applications. Incorporation of maleic acid and silane-treated coconut shell in PLA composites improved interfacial adhesion, thus reducing the area exposed to enzymatic hydrolysis resulting in lower biodegradation rate [29]. Further, the mechanical and thermal stability of coconut shell biomass-based composites improved with alkali treatment [30]. An investigation on the impact of bleaching processes on the chemical, physical, and functional properties of coconut shell revealed that H_2_O_2_ bleaching improves the water-holding capacity of the resultant composite [31]. This indicates that subjecting cellulosic materials to different chemical treatments results in different properties enhancement. Alkali and bleaching treatments have been reported as the most effective and popular surface modification techniques, resulting in enhanced natural fibre/matrix interfacial compatibility as well as microstructural modification of cellulosic materials [32,33]. However, limited to the authors’ literature survey, comparative studies on the effect of alkali, bleaching, and combined NaOH-bleaching treatments on surface morphology, thermal stability, and chemical characteristics of coconut shell biomass have seldom been investigated and reported.

The present study attempts to enhance the thermal stability, morphological, and chemical properties of coconut shell biomass through alkali, bleaching, and combined NaOH-bleaching surface treatments. The evaluated hypothesis was that the structural, thermal, and chemical properties of coconut shell biomass would be enhanced by surface chemical treatment for possible improved coconut shell/polymer matrix compatibility. The scope of the current study is to compare the properties of raw and treated coconut shell biomass subjected to different chemical solutions and establish the most effective surface treatment for non-cellulosic component elimination. The removal of amorphous constituents tends to improve the properties of the treated coconut shell biomass for potential polymer composite applications, thus offering various benefits, such as renewability, sustainability, environmental friendliness, low cost, and lightweight attributes over conventional synthetic materials. The novelty of this work lies in the value addition of coconut shell agricultural residues, thus converting waste to wealth.

## 2 Material and methods

### 2.1 Materials

Coconut shell was obtained from a farm plantation in Akure, Ondo State, Nigeria. The main chemical reagents used are sodium hydroxide (NaOH, 97 %), sodium chlorite (NaClO_2_, 80 %), absolute ethanol (C_2_H_5_OH, 99 %), sulphuric acid (H_2_SO_4_, 98 %), toluene (C_7_H_8_, 99.5 %), and glacial acetic acid (CH_3_COOH, 99.7 %).

### 2.2 Coconut shell biomass powder preparation

**Fig. 1:**
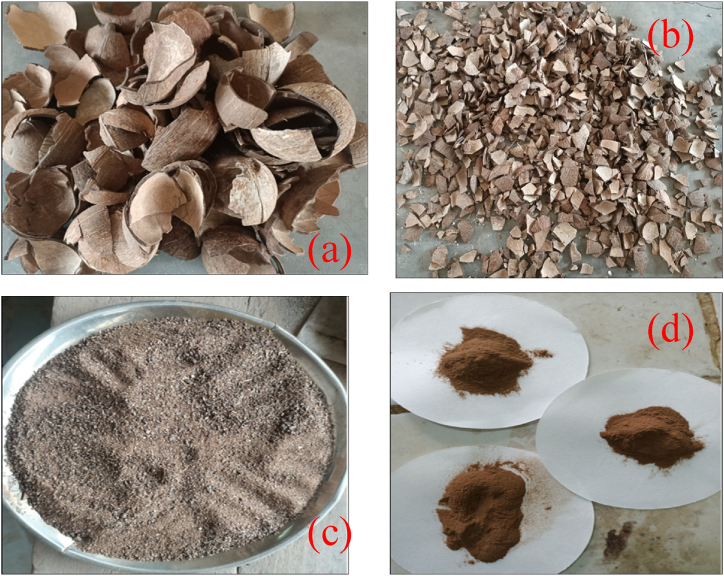
Pictorial representation of (a) Clean and Dry coconut shell, (b) crushed coconut shell, (c)Ball milled coconut shell, and (d) 150 μm ball-milled coconut shell biomass powder.

The kernel and other contaminants on coconut shell surfaces were removed using a knife scrapper and hard sandpaper. After that, the coconut shells were soaked in water for 24 h, followed by re-soaking in hot water at 60°C for 2 h to rinse the coconut shells and to facilitate the elimination of dirt and other impurities from the surface. The washed coconut shells were dried in a blast air oven (Model No. DHG-9101.1SA) at 105°C for 4 h, as depicted in Fig. 1(a). The coconut shells were crushed and milled using a laboratory crusher and ball milling machine, as presented in Fig. 1 (b) and 1 (c). The milled samples were sieved through 50–100 mesh sieve sizes (150–300 μm particle sizes), as presented in Fig. 1 (d). The powdered coconut shell was dried in a blast air oven at 105°C for 4 h, cooled in a desiccator, and stored at room temperature in airlock bags for further characterization and chemical treatments.

### 2.3 Surface treatment of coconut shell biomass

The coconut shell biomass was treated with sodium hydroxide (NaOH), sodium chlorite (bleaching), and combined NaOH-bleaching treatments. A constant mass-to-liquor ratio of 1:20 (g: mL), that is, for each 5 g of sample, 100 mL of the mixture solution was used for all chemical treatments of the coconut shell biomass conducted in this study.

#### 2.3.1 Alkaline treatment

**Fig. 2:**
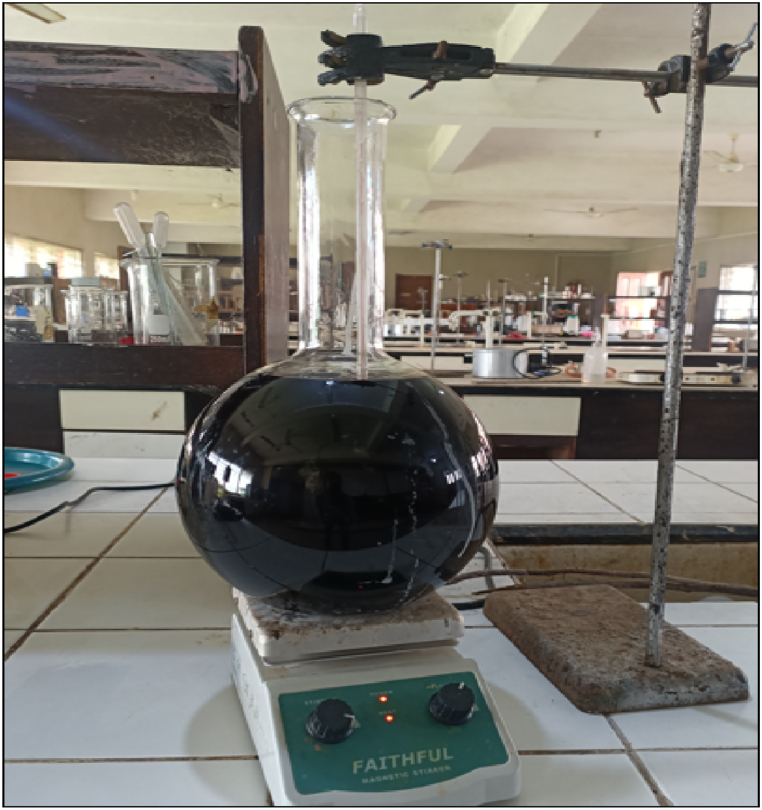
Chemical treatment of coconut shell biomass powder under mechanical stirring and controlled temperature.

Coconut shell biomass was treated with 4% (w/v) NaOH solution under constant mechanical stirring at 80°C for 4 h to eliminate hemicelluloses, which dissolve in the treatment solution. First, the sodium hydroxide solution was heated to 80°C while stirring constantly for 30 min before adding the sample. After that, the raw biomass powder sample was added to the pre-heated mixture solution followed by further heating for 4 h at this temperature under constant mechanical stirring using a magnetic stirrer (Huanghua Faithful Instrument Co., Ltd; Model: SH-2) as shown in Fig. 2. After the 4 h, the reaction was quenched by placing the reaction mixture in an ice bath to stop the treatment reaction and cooled to room temperature. The alkali-treated sample was washed and filtered using distilled water on a 100 μm wire cloth filter sheet until a neutral pH of 6–7 was attained. Finally, the alkali-treated sample was dried in a hot air-circulated oven at 105°C for 4 h, ground, sieved through 150 μm sieve size, and stored in airtight zip-lock polybags for further characterization.

#### 2.3.2 Bleaching treatment

For bleaching treatment, raw coconut shell biomass powder was treated using acetate buffer (2.7% (w/v) NaOH and 7.5% (v/v) glacial acetic acid), 1.7% (w/v) NaClO_2_, and distilled water of equal volume. The solution mixture was first heated to 80°C while stirring constantly for 30 min before adding the raw biomass sample. The sample was added to the pre-heated mixture solution, followed by further heating at this temperature for 4 h under constant mechanical stirring and controlled temperature, as illustrated in Fig. 2. After the 4 h, the reaction was quenched by placing the reaction mixture in an ice bath to stop the treatment reaction and cooled to room temperature. The bleach-treated sample was washed and filtered using distilled water on a 100 μm wire cloth filter sheet until a neutral pH of 6–7 was attained. The obtained white material, which is referred to as holocellulose, contains cellulose and hemicellulose. The bleaching treatment aimed to remove lignin and residual hemicellulose, which dissolve in the treatment solution. This whitens the sample by breaking down phenolic compounds, molecules, and chromophoric groups in lignin and removing the by-products. Finally, the bleach-treated sample was dried in a hot air-circulated oven at 105°C for 4 h, ground, sieved through 150 μm sieve size, and stored in airtight zip-lock polybags for further characterization.

#### 2.3.3 Combined NaOH-bleaching treatment

**Fig. 3:**
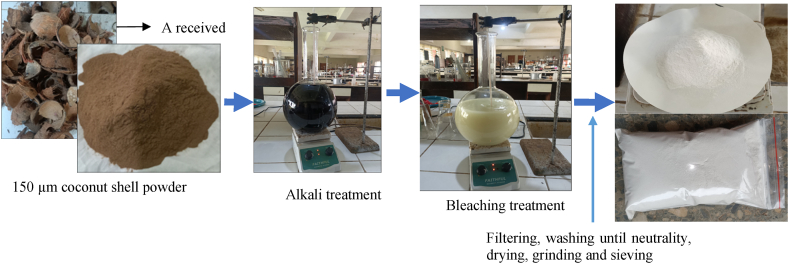
Schematic Flowchart for combined NaOH- bleaching treatment.

A two-step approach was adopted for combined NaOH-bleaching treatment. Firstly, raw coconut shell biomass was treated using NaOH solution, as explained in alkaline treatment. Thereafter, the alkali-treated coconut shell biomass was treated using a bleaching solution, as discussed in the bleaching treatment and presented in Fig. 3.

### 2.4 Chemical characterization

Chemical characterization was conducted to quantify the percentages of holocellulose, extractives, lignin, hemicellulose, moisture, cellulose, and ash content in raw and chemically modified coconut shell biomass following TAPPI standard methods. TAPPI T412 om-16 standard [34] was adopted in moisture content determination, whereas TAPPI T211 om-07 standard [35] was followed for ash content determination on a moisture-free sample. TAPPI T204 cm-07 standard [36] and TAPPI T222 om-06 standard [37] procedures were adopted in extractives and acid-insoluble lignin content determination, respectively. On the other hand, the procedures proposed by Wise and Ratliff [38] and TAPPI standard T203 cm-99 [39] procedures were followed in quantifying holocellulose and cellulose proportions, respectively. The difference between holocellulose and cellulose was used to determine the hemicellulose content with the help of Equation (1).

(1) Hemicellulose%=holocellulose%−cellulose%

All the chemical constituents were analyzed in triplicates, and the average readings were reported.

### 2.5 Functional group investigation

The various functional groups in raw and chemically modified coconut shell biomass were identified using a Fourier transform infrared instrument (FT-IR 8400S). The samples were coated with potassium bromide (KBr) before analysis on an FTIR machine operated at 32 scans/minute scanning rate using 2 cm^−1^ signal-to-noise ratio resolution. The readings were recorded in a region from 4000 to 400 cm^−1^.

### 2.6 X-ray diffraction (XRD) analysis

The crystallographic properties of raw and chemically modified coconut shell biomass were determined using the XRD analysis technique using a Malvern Panalytical Aeris XRD diffractometer working at a scanning rate of 0.02° per minute and a scattering 2θ angle range of 5°–80° with radiation of 1.79206 Å at ambient conditions. Following the Segal method [40], the crystallinity index of the samples was calculated using Equation (2).

(2) $$CrI.\%=\frac{I_{200}-I_{am}}{I_{200}}\;x\;100$$

where; *I*_200_ and *I*_*am*_ represent the maximum peak intensity of the crystalline phase (2θ = 22–26°) and the intensity of the amorphous phase (2θ = 18–20°), respectively.

The Debye-Scherrer's equation (Equation (3)) was used to determine the crystallite size (D) of the samples [41].

(3) $$D=\frac{0.91\;x\;0.179206}{\beta_{1/2}\;\cos\;\theta }$$

where; β_1/2_ = full width at half maximum (FWHM) of the XRD peak in radians and θ (half of 2θ) is the corresponding Bragg's angle. Equation (4) was used to compute FWHM (β_1/2_) in radians.

(4) $$\beta_{1/2}=\frac{FWHM\;x\;\pi }{180}$$

The percentage crystalline (P.C.) of the samples was determined using Equation (5) [42].

(5) $$Degree\;of\;crystallinity\;or\;P.C.\%=\frac{\;I_{cr}}{\;I_{cr}+I_{am}\;}\;x\;100$$

Where I_200_ and I_am_ are the same as Equation (2).

### 2.7 Thermogravimetric analysis (TGA)

The thermograms of raw and chemically-treated coconut shell biomass were recorded using a PerkinElmer/TGA4000 thermogravimetric analyzer (TGA) instrument at a heating rate of 10 °C per minute from room temperature to 860 °C under N_2_ atmosphere [43].

### 2.8 Morphological characterization

#### 2.8.1 Scanning electron microscopy (SEM)

The transformation of surface morphology due to chemical treatments was analyzed using the Scanning Electron Microscope (ZEISS EVO 18) with an accelerated voltage of 15 kV.

#### 2.8.2 Energy dispersive X-ray spectroscopy (EDX)

The presence of elements, like carbon, oxygen, calcium, aluminium, Sulphur, etc. on the surface of untreated and chemically treated coconut shell biomass was detected and quantified using an EDX instrument attached to the SEM microscope.

## 3 Results and discussion

### 3.1 Chemical constituents

The structure of a cellulosic material consists of crystalline and amorphous components. The former comprises crystalline cellulose proportions, while the latter contains amorphous constituents such as wax, pectin, hemicellulose, extractives, lignin, etc. The presence of amorphous components on the surface of a cellulosic material adversely affects its compatibility with hydrophobic polymeric matrices. As a lignocellulosic material, coconut shell agricultural residue contains hemicellulose, cellulose, and lignin as the principal chemical elements. Cellulose accounts for the strength and stability of the cell walls, hence enhancing tensile strength, crystallinity index, stiffness, and thermal characteristics of lignocellulosic fibres [44]. A higher cellulose content indicates an improved performance of cellulosic fibres when used as reinforcing materials in polymeric matrices. Hemicellulose accounts for the biodegradation, water uptake, and thermal decomposition properties of natural fibres. Conversely, lignin accounts for the thermal stability, water sorption, flexibility, morphological, and structural properties of cellulosic biomass [45]. Table 1 presents the chemical composition (in percentage) of components in untreated and treated samples.

**Table 1:** Effect of chemical treatment on chemical composition of coconut shell.

Treatment	Chemical composition %					
	Cellulose	Hemicellulose	Lignin	Extractives	Moisture	Ash
Raw	30.22 ± 0.22	10.13 ± 0.09	47.13 ± 0.11	4.51 ± 0.07	5.6 ± 0.16	2.36 ± 0.10
Alkaline-	49.93 ± 0.19	5.25 ± 0.21	34.54 ± 0.54	3.23 ± 0.04	4.39 ± 0.12	2.59 ± 0.09
Bleaching-	52.89 ± 0.08	6.98 ± 0.05	29.38 ± 0.33	3.67 ± 0.10	4.12 ± 0.19	2.86 ± 0.06
NaOH-Bleaching	74.72 ± 0.10	4.01 ± 0.16	11.62 ± 0.39	2.83 ± 0.15	3.79 ± 0.09	2.95 ± 0.07

Table 1 shows that untreated coconut shell biomass has a higher percentage of non-cellulosic contents (lignin, hemicellulose, ash, extractives, etc.) and a lower percentage of cellulose content. However, chemical modification of coconut shell through alkaline, bleaching, and combined NaOH-bleaching treatment considerably increased the cellulose content while the percentage of non-cellulosic content drastically reduced. This observation can be ascribed to the partial elimination of amorphous constituents of hemicellulose and lignin regions through hydroxylation and depolymerization, respectively, as confirmed by FTIR investigation [46]. Further, it can be observed that a combination of NaOH-bleaching treatment resulted in a higher cellulose content increment, indicating the effectiveness of the combined chemical treatment in the fractional elimination of amorphous constituents. This observation is congruent and consistent with a study by Akindoyo et al. [47] that reported a higher cellulose content for combined alkali and bleaching treatment compared with alkali treatment.

### 3.2 FTIR spectral analysis

**Fig. 4:**
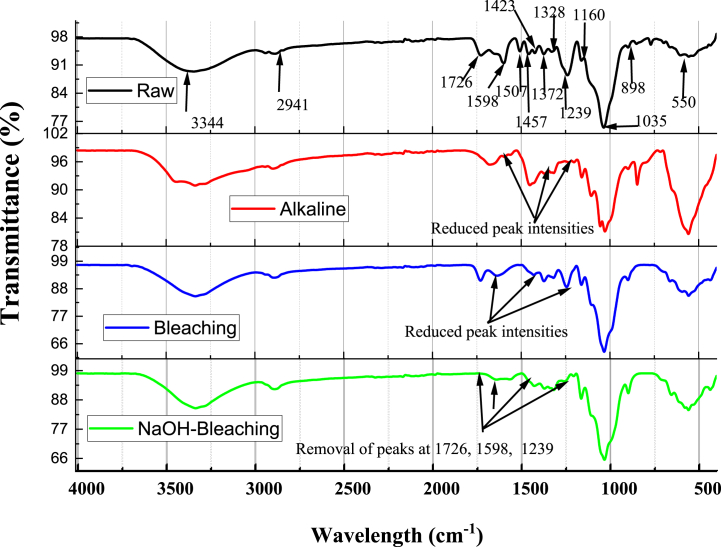
FTIR Spectra of Untreated, Alkaline-, Bleach-, NaOH-bleach-treated coconut shell agricultural biomass powder.

Fig. 4 displays the functional groups in terms of infrared peaks of untreated and chemically treated coconut shell agricultural residue biomass powder. The untreated sample showed peaks at 3344 and 2941 cm^−1^ ascribed to stretching and vibration of O-H and C-H confirming the presence of α-cellulose, water, and alcohol; and hemicellulose, cellulose, and other organic compounds presence, respectively [48,49]. The peak recorded at 1726 cm^−1^ is ascribed to strong carbonyl (C=O) stretching vibrations of acetyl groups in lignin, hemicellulose, and pectin. Also, C=C aromatic symmetric stretch vibrations with strong conjugated C–C bonds observed at 1598 cm^−1^ denote lignin concentration [50]. The peak at 1457 cm^−1^ indicates lignin presence due to stretch vibrations of C–H functional groups [51]. The peaks recorded at 1372 cm^−1^ and 1239 cm^−1^ are due to symmetric and asymmetric stretch vibrations of C–H functional groups denoting hemicellulose presence and stretch vibration of C = O and O – C – O groups of lignin, respectively. A strong peak at 1035 cm^−1^ depicts asymmetric stretch vibrations of O–C–O ester groups in cellulose [50]. In contrast, a small peak at 898 cm^−1^ represents the β-glycosidic linkage between the monosaccharides in raw lignocellulosic sources associated with skeletal C=C bending of cellulose [52].

The FTIR spectrograms of alkali- and bleach-treated samples are relatively similar to those of untreated counterparts except for diminishing peak intensity around the peaks at 3344, 1726, 1598, 1423, 1328, and 1239 cm^−1^. In the case of the combined NaOH-bleach-treated sample, there was complete removal (or disappearance) of peaks at 1726, 1598, 1457, 1328, and 1239 cm^−1^ observed in the raw sample. This observation implies that combined NaOH-bleaching treatment effectively removes non-cellulosic contents from the surface of the coconut shell. Moreover, the peak at 1035 and 898 cm^−1^ for the raw sample reported similar peak intensities even after chemical treatments. This indicates that cellulose structure does not degrade (or decompose) under chemical treatment due to its higher molecular weight compared with lignin and hemicellulose [53], as illustrated in Table 2. Instead, chemical treatments increase cellulose proportion by decreasing the proportions of non-cellulosic constituents, as shown in the chemical constituent analysis. A similar trend for the influence of chemical modification has been reported for *Bauhinia vahlii* bast fibre subjected to alkaline, bleaching, and benzoylation treatments [22].

**Table 2:** FTIR peak locations, functional groups, and corresponding fibre components in raw and chemically treated coconut shell powder.

Peak position (wavelength cm^−1^)				Corresponding functional groups	Fibre component	References
Raw	Alkali	Bleach	NaOH-bleach			
3344	3344^a^	3344^a^	3344	O–H stretching	Cellulose	[49]
2941	2941	2941	2941	C–H stretching vibration	Hemicellulose, cellulose	[48]
1726	1726^a^	1726^a^	—	Strong carbonyl (C=O) stretching	Hemicellulose, lignin	[54]
1598	1598^a^	1598^a^	—	C=C aromatic stretch vibration;	Lignin	[50]
1457	1423^a^	1423^a^	—	C–H stretching	Lignin	[51]
1372	1328	1328	—	C–H symmetric and asymmetric stretching	Hemicellulose	[50]
1239	1239^a^	1239^a^	—	C = O and O–C–O	Lignin/hemicellulose	
1035	1035	1035	1035	O–C–O asymmetric stretch vibration	Cellulose	
898	898	898	898	β-glycosidic rings	Cellulose	[52]

^a^ = Peak removal/disappearance.

— = Reduced peak intensity.

### 3.3 XRD analysis

**Fig. 5:**
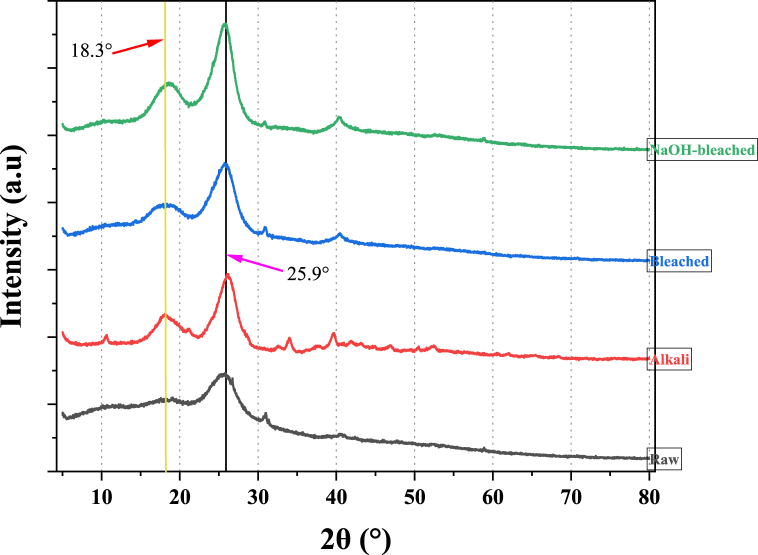
X-ray diffraction patterns of raw and chemically modified coconut shell biomass.

Fig. 5 depicts the XRD patterns of raw and chemically modified coconut shell biomass with two major peaks at 2θ = 18.3° and 25.9° and a small peak at 2θ ≈ 40°. These peaks correlate to (110), (200) and (004) lattice planes, respectively, for cellulose I crystallites. The peak around 18.3° is referred to as an amorphous peak, denoting the presence of amorphous components like lignin, pectin, wax, hemicellulose, etc. In contrast, the crystalline peak around 25.9° indicates the presence of crystalline components [43,55]. Conversely, the peak at about 40° designates the presence of inorganic compounds like aluminium (Al), calcium (Ca), and silicon (Si), among others [56], as revealed in EDX analysis.

Fig. 5 shows that the peak at 2θ = 25.9° becomes sharper and amplified after surface modification of the samples, indicating enhancement of crystallographic properties of the materials, as illustrated in Table 3.

**Table 3:** Effect of chemical treatments on crystallographic properties of coconut shell.

Sample	2θ (200) (°)		2θ (Amorphous) (°)		CrI.%	% Crystalline	Crystallite size (nm)
	Degree	Intensity (I_200_)	Degree	Intensity (I_am_)			
Raw	25.51	2208.85	20.84	1218.86	44.82	64.44	3.84
Alkali	25.50	4772.61	21.56	1354.48	71.62	77.89	3.58
Bleach	25.56	3347.05	21.28	1104.48	67.00	75.19	5.06
NaOH-bleach	23.84	1134.94	17.55	223.72	80.29	83.53	1.97

The crystallinity indices, crystallite sizes, and percentage crystalline of the raw and chemically modified samples as computed using Equations (2), (3), (5), respectively, are presented in Table 3. The table shows an enhancement in crystallinity index after chemical modification with combined NaOH-bleach treated samples, reporting the highest crystallinity index of 80.29 %. Generally, the crystallinity index follows the order of NaOH-bleaching > alkali > bleaching > untreated. Similarly, the study observed an increase in percentage crystalline (P. C) with surface modification. The observed trend can be credited to the partial elimination of amorphous constituents through chemical treatments, as demonstrated in the FTIR analysis. This allows cellulose to assume a more crystalline form. Similar results on the influence of chemical modification have been reported for *Furcraea foetida* leaf fibre [57] and *Bauhinia vahlii* bast fibre subjected to different surface treatments [22]. However, the study observed mixed findings on the influence of chemical treatments on crystallite size. For instance, bleaching treatment improved the sample crystallite size to 5.06 nm compared to 3.84 nm for raw sample. Similar trend of increasing crystallite size with chemical treatment has been reported for alkali-treated *Grewia Flavescens* natural fibre (68.43 nm) compared to 62.90 nm for raw fibre [58]. This observation can be attributed to the partial removal of amorphous proportions, thus increasing the amount of crystalline form of α-cellulose. On the other hand, alkali and combined NaOH-bleaching treatments reduced crystallite size with reference to untreated samples. This phenomenon can be attributed to the possibility of a reaction of chemical reagents with the chain ends of the crystalline region. It is difficult for chemical reagents to disperse to the crystalline region, making hydrogen-bonded cellulose chains to open, forcing the reagents to disperse to the newly produced amorphous cellulose [59]. This generates more amorphous cellulose, thus explaining the observed reduction in average crystallite size. Similarly, a reduction in crystallite size as a result of chemical treatment has been reported for alkali- (12.81 nm) and NaOH-bleach-treated (10.12 nm) compared to 16.30 nm for untreated Z*iziphus jujuba* fibres [60].

### 3.4 Thermal analysis

**Fig. 6:**
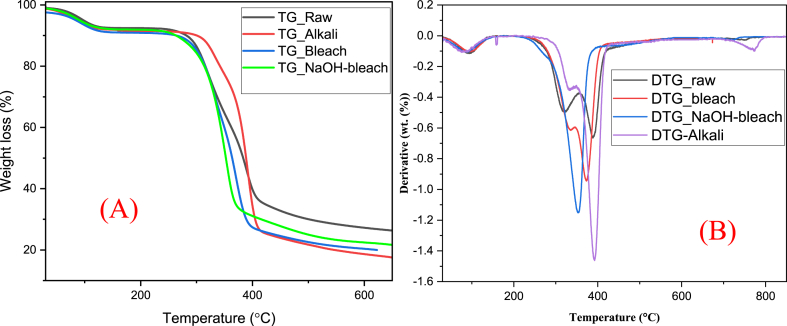
Graph of (A) TG Thermograms and (B) DTG thermograms of raw and chemically-modified coconut shell agricultural biomass.

Fig. 6 (A) and 6 (B) show the thermal behaviour of raw and chemically modified coconut shell biomass, with the former exhibiting four thermal degradation stages. From room temperature to 100°C, raw and chemically treated samples underwent an initial degradation phase with a minimal weight loss (<10%), representing the evaporation of water and moisture present in cellulosic materials as temperature increases.

The second degradation stage, which represents hemicellulose decomposition, took place at a temperature range of 250–323°C for raw, 255–336°C for alkali-treated, and 134–238°C for bleach-treated samples with an associated weight loss of 13.98, 23.72, 8.06, and 27.70% for raw, alkali, bleached, and NaOH-bleach, respectively. Alkali treatment of coconut shell biomass reported an increase in onset temperature of degradation to 255°C from 250°C for raw samples. This indicates an improvement in thermal stability for the alkali-treated sample compared to the untreated coconut shell. The enhanced thermal stability of alkali-treated samples can be attributed to the partial elimination of highly volatile and low molecular mass compounds, mainly fatty acids, extractives, lipids, hemicellulose, etc. However, bleaching and combined NaOH-bleaching treatments reported reduction in onset temperature of degradation to 134°C and 232°C, respectively, compared to 250°C for raw sample due to excessive elimination of lignin constituents that are responsible for the thermal behavior of lignocellulosic materials.

In the third phase of degradation, maxima degradation peaks were recorded at 389°C for raw, 373°C for alkali-treated, 291°C for bleach-treated, and 352°C for NaOH-bleach-treated samples. The phase represents cellulose degradation with associated weight loss of 41.43% for raw, 17.79% for alkali-treated, and 39.69% for bleach-treated samples. The last phase of degradation corresponding to lignin decomposition occurred at temperatures above 400°C for all samples with an associated percentage weight loss of 23.89% for raw, 11.04% for alkali-, 7.99% for bleach-, and 14.26% for NaOH-bleach samples. Investigation of the thermal behaviour of *Bauhinia vahlii* fibre subjected to different surface treatments reported similar findings [22]. Generally, from Fig. 6(A), it can be noted that the TG bleach curve stopped at 620°C yet all samples were heated up to 860°C, indicating a possible excessive delignification of the sample, causing extreme lignin decomposition.

### 3.5 Surface morphological analyses

#### 3.5.1 Scanning electron microscopy

**Fig. 7:**
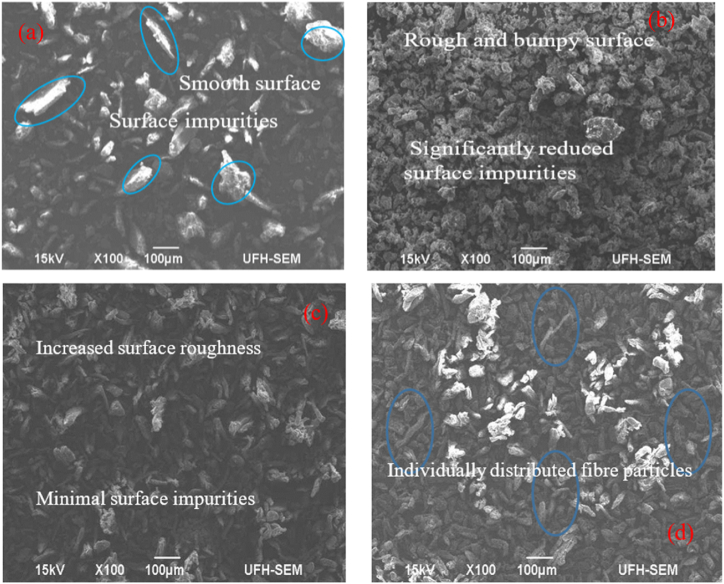
SEM micrographs of coconut shell: (a) Untreated, (b) alkali-, (c) bleach- and (d) NaOH-bleach-treated coconut shell.

The surface morphology of untreated samples was analyzed as a reference for the surface morphologies of chemically treated samples. Fig. 7 depicts the SEM micrographs of raw and treated samples. Fig. 7 (a) reveals a smooth and even surface with contaminants and impurities like wax, lignin, etc., on the untreated sample surface. Fig. 7 (b) depicts a rough and bumpy surface with significantly reduced surface impurities due to alkali treatment that facilitates the elimination of hemicellulose, wax, pectin, and other contaminants. A study on the effect of alkali treatment on the performance characterization of Ziziphus mauritiana fibre observed that alkali treatment eliminated a significant proportion of amorphous constituents and subsequently enhancing its surface roughness [61]. For bleach-treated samples (Fig. 7(c)), the study noted a similar observation of increased surface roughness and reduced surface impurities. This is because bleaching treatment further advances the partial elimination of surface contaminations, impurities, and other non-cellulosic regions, particularly hemicellulose and lignin, besides breaking the bond between them, as shown in (Fig. 7 (c)). Combined NaOH-bleaching treatment resulted in further breakdown of the complex lignocellulosic structure and dissolution of hemicellulose and lignin. This exposes additional porosity and increases the available surface area for concealed cellulose. This makes cellulosic fibres aligned and individually distributed, as illustrated in Fig. 7 (d). Therefore, from the analysis, chemical treatment enhances the surface roughness of the coconut shell, indicating improvement in mechanical interlocking. This is expected to improve compatibility between cellulosic fibre and polymer matrix; thus, better fibre/matrix interfacial bonding strength, enhancing the mechanical performance of the resultant bio-composites [62]. Removal of surface impurities and increase in surface roughness due to alkali and bleaching treatments have been reported for *Kigelia Africana* cellulosic fibre [63].

#### 3.5.2 Energy-dispersive X-ray (EDX) spectroscopy

**Fig. 8:**
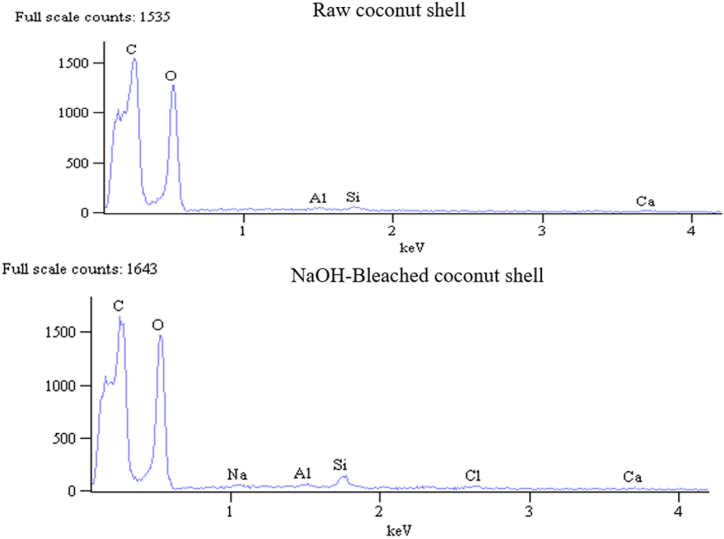
EDX Spectra of Raw and NaOH-bleach treated coconut shell.

As cellulosic materials, raw and chemically modified samples constitute higher oxygen (O) and carbon (C) proportions than any other element. This is because C and O are the primary elements in the cellulose chemical chain assembly, which is the main chemical constituent of coconut shell, as revealed in the chemical constituents analysis. Besides carbon and oxygen, the raw sample reported some trace elements of aluminium (Al), Calcium (Ca), and silicon (Si) obtained by the plant from the soil through roots, as shown in EDX spectra (Fig. 8) and tabulated in Table 4.

**Table 4:** Elemental composition and C/O ratio of raw and chemically-treated coconut shell.

Sample	Elemental composition (%)							
	C	O	Na	Al	Cl	Ca	Si	C/O
Raw	36.19 ± 1.52	56.95 ± 0.78	_	0.50 ± 0.09	_	0.77 ± 0.14	0.94 ± 0.09	0.64
Alkali-	40.14 ± 1.45	54.47 ± 0.8	2.04 ± 0.26	_	_	_	1.98 ± 0.2	0.74
Bleach-	43.71 ± 1.3	50.25 ± 0.74	2.34 ± 0.21	1.1 ± 0.09	_	_	2.59 ± 0.18	0.87
NaOH-bleach	45.98 ± 1.72	49.48 ± 0.74	0.53 ± 0.1	0.56 ± 0.08	0.8 ± 0.11	0.5 ± 0.13	2.15 ± 0.16	0.93

Further, treated samples showed the presence of sodium (Na) and chlorine (Cl) trace elements that were not detected in untreated samples due to residue impurities from alkali and bleaching treatments, respectively. Similarly, minor impurities of Na and Cl due to alkali and bleaching treatments of different agricultural residues have been reported in literature [[64], [65], [66]]. Therefore, besides ratifying the compositional constituents of cellulosic fibres, EDX analysis confirms the existence of some new elements due to chemical treatments. The study observed an increase in carbon percentage content and a decline in oxygen proportion from the raw sample to the combined NaOH-bleach-treated sample, as shown in Table 4. Consequently, the C/O ratio increased from 0.64 for the raw sample to 0.74 for alkali-, 0.87 for bleach-, and 0.93 for NaOH-bleach treated samples. This indicates enhanced hydrophobicity of the fibre surface, a desirable characteristic in developing plant fibre-based composites with enhanced characteristics. An increase in the C/O ratio, hence hydrophobicity with chemical treatment, can be ascribed to the fractional elimination of amorphous regions responsible for the hydrophilic nature of plant fibres. The literature survey shows an increase in the C/O ratio from 1.25 for raw to 1.59 for isocyanate-treated cellulose pulp fibre [67].

## 4 Potential applications

Polymer composites are the intended application area of raw and chemically modified coconut shell biomass investigated in this study. Due to its light density, renewability, sustainability, biodegradability, and low-cost attributes, raw and chemically modified coconut shell biomass have high potentiality in the manufacture of lightweight composites for structural and non-structural applications, such as automobile, food packaging, etc. This will provide lightweight, low-cost, sustainable, high abundance, and environmentally friendly substitute for synthetic fibres in engineering applications. Thus presenting a sustainable solution to unsustainability and nonbiodegradability challenges responsible for the global environmental pollution. The high cellulose content reported in the current study indicates that coconut shell biomass can be potentially used for nanocellulose extraction. The extraction of nanocellulose, with enhanced mechanical and thermal properties as a result of increased surface area can be used as potential fillers in polymeric composite fabrication. This will diversify the areas of application of coconut shell biomass for various engineering applications. Thus, making significant contributions towards coconut shell agricultural waste value addition and improved livelihoods and environmental health.

## 5 Conclusions

The chemical, thermal, structural, and morphological properties of raw and chemically-treated coconut shell biomass were investigated for potential polymer composite applications. Alkali, bleaching, and combined NaOH-bleaching treatments facilitated partial removal of non-cellulosic/amorphous constituents from the fibre surface, resulting in increased surface purity and roughness, cellulose constituents, thermal stability, and crystallinity index. As confirmed by FTIR, the contents of non-cellulosic components (lignin, hemicellulose, wax, etc) significantly decreased post-chemical treatment, indicating an increase in biomass hydrophobicity for better filler/matrix interlocking. SEM morphological investigation revealed improved surface roughness due to partial elimination of amorphous constituents. Coconut shell biomass subjected to combined NaOH-bleaching treatment exhibited maximum cellulose content (74.72 %) and crystallinity index (80.29 %) whereas alkali-treated biomass reported an increase in onset temperature of degradation, an indication of improved thermal stability. Based on the findings of the present study, it can be concluded that combined NaOH-bleach treatment is the best surface treatment method for non-cellulosic constituent removal as it presents excellent chemical, structural, and morphological properties. Therefore, there is a need for near-future studies to explore the synergistic effects of combining chemically-treated coconut biomass with other cellulosic fibres to develop hybrid composites with enhanced properties for diverse engineering applications.

## CRediT authorship contribution statement

**Dennis O. Bichang'a:** Writing – review & editing, Writing – original draft, Validation, Resources, Methodology, Investigation, Formal analysis, Data curation, Conceptualization. **Isiaka O. Oladele:** Writing – review & editing, Writing – original draft, Supervision, Methodology, Investigation, Formal analysis, Data curation, Conceptualization. **Oladunni O. Alabi:** Writing – review & editing, Supervision, Resources, Conceptualization. **Fatai O. Aramide:** Supervision, Resources, Conceptualization. **Olasunkanmi Oluseye:** Methodology, Formal analysis, Data curation. **Sunday G. Borisade:** Writing – review & editing, Methodology, Investigation, Formal analysis. **David N. Githinji:** Writing – review & editing, Validation, Resources. **Mike O. Ojemaye:** Writing – review & editing, Validation, Resources, Data curation.

## Data availability

The corresponding author will provide the data on request.

## Funding declaration

The authors did not receive any financial or non-financial support from any organization for the submitted work.

## Declaration of competing interest

The authors declare that they have no known competing financial interests or personal relationships that could have appeared to influence the work reported in this paper.
